# Evaluation of Subclinical Hypothyroidism in Children and Adolescents: A Single-Center Study

**DOI:** 10.1155/2016/1671820

**Published:** 2016-07-27

**Authors:** Kamila Such, Aneta Gawlik, Aleksandra Dejner, Malgorzata Wasniewska, Agnieszka Zachurzok, Aleksandra Antosz, Tomasz Gawlik, Ewa Malecka-Tendera

**Affiliations:** ^1^School of Medicine in Katowice, Medical University of Silesia, Medical Students' Scientific Association, Department of Pediatrics and Pediatric Endocrinology, Ulica Medykow 16, 40-752 Katowice, Poland; ^2^School of Medicine in Katowice, Medical University of Silesia, Department of Pediatrics and Pediatric Endocrinology, Ulica Medykow 16, 40-752 Katowice, Poland; ^3^Department of Pediatric, Gynecological, Microbiological and Biomedical Sciences, University of Messina, Via Consolare Valeria, 98125 Messina, Italy; ^4^Upper-Silesian Pediatric Health Center, Department of Pediatrics and Pediatric Endocrinology, Ulica Medykow 16, 40-752 Katowice, Poland; ^5^Department of Nuclear Medicine and Endocrine Oncology, Maria Sklodowska-Curie, Memorial Cancer Center and Institute of Oncology, Gliwice Branch, Ulica Wybrzeze Armii Krajowej 15, 44-400 Gliwice, Poland

## Abstract

The main purpose of our retrospective study was to evaluate the medical care of the patients with subclinical hypothyroidism (sHT) and to investigate the rationale for administering L-thyroxine (LT-4) to young sHT patients.* Patients and Methods*. Based on a retrospective review of the charts of 261 patients referred to the Endocrinology Outpatient Clinic between 2009 and 2014 with suspicion of sHT, 55 patients were enrolled for further analysis. Data collected was baseline age, anthropometric measurements, serum TSH, fT4, fT3, anti-thyroid autoantibodies, positive family history, absence/presence of clinical symptoms, length of follow-up, and data concerning LT-4 therapy (therapy: T1; no therapy: T0).* Results*. T1 encompassed 33 (60.0%) patients. There were no differences between T1 and T0 (*p* > 0.05) with regard to age, TSH concentrations, BMI *Z*-score, and hSDS values, though follow-up was longer in T1 (*p* < 0.01). Four (11.8%) children in T1 and none in T0 had a positive family history of thyroid disorders. Fifteen (68.2%) patients in group T0 became euthyroid. One (1.8%) girl (T1) developed overt hypothyroidism.* Conclusions*. A small percentage of patients can proceed to overt hypothyroidism. Only positive family history seemed to influence the decision to initiate LT-4 therapy. Further prospective studies are warranted in order to establish treatment indications, if any, and the mean recommended dosage of LT-4.

## 1. Introduction

The term subclinical hypothyroidism (sHT) indicates the presence of thyroid dysfunction without obvious symptoms of thyroid deficiency [1]. This means that the disorder may be at an early stage of evolution [2]. However, the clinical manifestations of sHT vary widely, from minimal, nonspecific (often subjective), or even absent signs, to clear presentation of hypothyroidism, largely related to the degree of deterioration of the thyroid gland, disease duration, and individual sensitivity to thyroid hormone deficiency, as well as the presence of comorbidities, especially heart and peripheral arterial diseases and depression. In adults, sHT is known to be associated with an increased risk of coronary and other heart diseases, as well as various biochemical disturbances (e.g., elevated LDL-cholesterol) [3, 4]. In children and adolescents, subtle proatherogenic abnormalities were found in only one study, conducted by Cerbone et al., and required a further scrupulous observation towards metabolic disorders [5].

In the clinical practice, sHT is diagnosed in the presence of mildly elevated serum concentrations of TSH with normal concentrations of serum-free and total triiodothyronine (T3) and thyroxine (T4) [6]. sHT is common in the adult population, with a prevalence of 3 to 8% in patients without known thyroid disorder [7, 8]; it tends to be higher in the elderly [9] and in women [7]. In the pediatric population, sHT is estimated to affect less than 2% according to some studies [3]. The main complication of sHT, both in adults and children, is progression to overt hypothyroidism, which may actually occur considerably less frequently than expected [10].

The 2001 review of studies in adult and elderly patients showed that although subclinical hypothyroidism is mild thyroid gland failure, it is clinically important, with adverse clinical consequences, and should be treated with L-thyroxine in most, if not all, cases [11]. Data concerning the younger population are limited. Most of the available longitudinal studies on the natural course of subclinical hypothyroidism in children and adolescents are retrospective. The published data are controversial and do not offer a conclusive solution regarding the reasons, if any, for treatment of subclinical thyroid disease in younger population. Thus the debate on the indications for L-thyroxine therapy in children and adolescents with sHT continues.

The aim of our retrospective observational study was to evaluate the course of subclinical hyperthyrotropinemia in children and adolescents and estimate the medical care of the patients with sHT: the rationale for administering L-thyroxine to young patients with sHT was investigated.

## 2. Patients and Methods

The study methodology was based on a retrospective review of the charts of 261 patients. The patients were referred to the Endocrinology Outpatient Clinic of the Medical University with suspicion of subclinical hypothyroidism and were examined between 2009 and 2014. The study was performed in a non-iodine-deficient area. Children and adolescents diagnosed with subclinical thyroid disease who were under concomitant therapy with lithium salts, antiepileptic agents, glucocorticoids, or iodinated drugs, as well as children with positive screening for congenital hypothyroidism, were not enrolled into the study. Other exclusion criteria were (1) serum concentration of thyroid-stimulating hormone (TSH) below <4 mIU/mL or above >10 mIU/mL, (2) Hashimoto's thyroiditis, (3) trisomy 21 and/or diabetes mellitus, (4) earlier treatment of previously diagnosed SH, (5) one/incidental finding of elevated TSH level between 4.0 and 10.0 mIU/mL, (6) palpable goiter, (7) age under 2 years, and (8) lack of anthropometric data.

### 2.1. Measurements

We collected the following data: gender, age at presentation of subclinical hypothyroidism (sHT), auxological data: height and weight, serum concentrations of thyroid-stimulating hormone (TSH), free thyroxine (fT4), and free triiodothyronine (fT3), titer of anti-thyroid autoantibodies: antiperoxidase (anti-TPO) and antithyroglobulin (anti-TG), positive family history, absence or presence of clinical symptoms suggesting thyroid disease, and length of follow-up at the Outpatient Clinic. Serum concentrations of TSH and fT4 were measured with a chemiluminescent immunometric assay (Siemens, Immulite 2000 Free T4, Immulite 2000 Third-Generation TSH, USA). The concentrations of anti-thyroid peroxidase antibody (anti-TPO Abs) and autoantibodies to thyroglobulin (anti-TG Ab) were determined with enzyme-labeled, chemiluminescent sequential immunometric assay (Siemens, Immulite 2000 anti-TPO Ab, Immulite 2000 Anti-TG Ab, USA). The reference levels for fT4 were 11.5–22.7 pmol/L (0.8–1.9 ng/dL). Anti-TG Abs and anti-TPO Abs were considered undetectable below 40 and 35 IU/mL, respectively. Thyroid ultrasound examination was performed at least once during the follow-up period, using Acuson Antares (Siemens Medical Solution USA, Inc.) with a VFX 13-5 linear transducer. Diffuse low echogenicity was considered an indicator of a thyroid autoimmune disorder. Subclinical hypothyroidism was diagnosed in patients with no evident clinical manifestation of hypothyroidism, regardless of their age, if TSH was between 4.0 and 10.0 mIU/mL in at least two consecutive assays and fT4 remained within the normal range. Overt hypothyroidism was diagnosed if TSH was higher than 10.0 mIU/mL and fT4 was below the lower limit of the reference range. Thyroid autoimmunity was documented by the presence of thyroid autoantibodies (anti-TPO Abs and/or anti-TG Abs). Hashimoto's thyroiditis (autoimmune thyroid disease) was diagnosed in the presence of anti-TPO Abs and/or anti-TG Abs, with concomitant subclinical or overt hypothyroidism.

A standard stadiometer was used to measure the patients' height. hSDS (height standard deviation scores) were calculated from population standards for healthy children using the following formula: hSDS = child's height − height for 50 pc/0.5 *∗* (height 50 pc − height 3 pc). Short stature was defined as hSDS below −2.0 standard deviation (SD). The results in the paper and tables are presented as mean ± SD.

Body mass index (BMI) was expressed as kg/m^2^, whilst BMI *Z*-score, also called BMI standard deviation (SD) score, was calculated using measures of relative weight adjusted for age and sex. Given a child's age, sex, BMI, and the appropriate reference standard, the BMI *Z*-score was calculated using The Pediatric *Z*-Score Calculator. The tool is available at the website of The Children's Hospital of Philadelphia, Research Institute http://stokes.chop.edu/web/zscore/ and is dedicated to patients aged between 2 and 20 years. A BMI *Z*-score over +2.0 SD was classified as obesity, between +2.0 and +1.0 SD as overweight, between −1.0 and −2.0 as weight deficiency, and under −2.0 SD as significant weight deficiency. The data in the paper and tables are presented as mean ± SD.

Clinical presentations suggesting subclinical hypothyroidism, such as constipation, impaired concentration, cold intolerance, chronic fatigue, menstrual disorder, dry skin, and hair loss, were detected at physical examination or during anamnesis.

A cumulative dose of L-thyroxine was expressed as a mean dosage, using the recommended amount of LT-4 on the first and last available visit in relation to body weight and was presented as *μ*g per kg per day.

### 2.2. Statistical Analysis

Statistical analysis was performed using PQ Stat software. Categorical variables were presented as percentages (%); continuous variables were ordered as means ± standard deviations (SD) and medians. The distribution plot was verified using Lilliefors test. The comparison between two groups of categorical variables was examined with Fisher's test. The comparison between continuous variables was examined with Student's *t*-test for normal distribution and with the nonparametric Mann-Whitney test for nonnormal distribution. A *p* value of less than 0.05 was considered statistically significant.

## 3. Results

**Figure 1:**
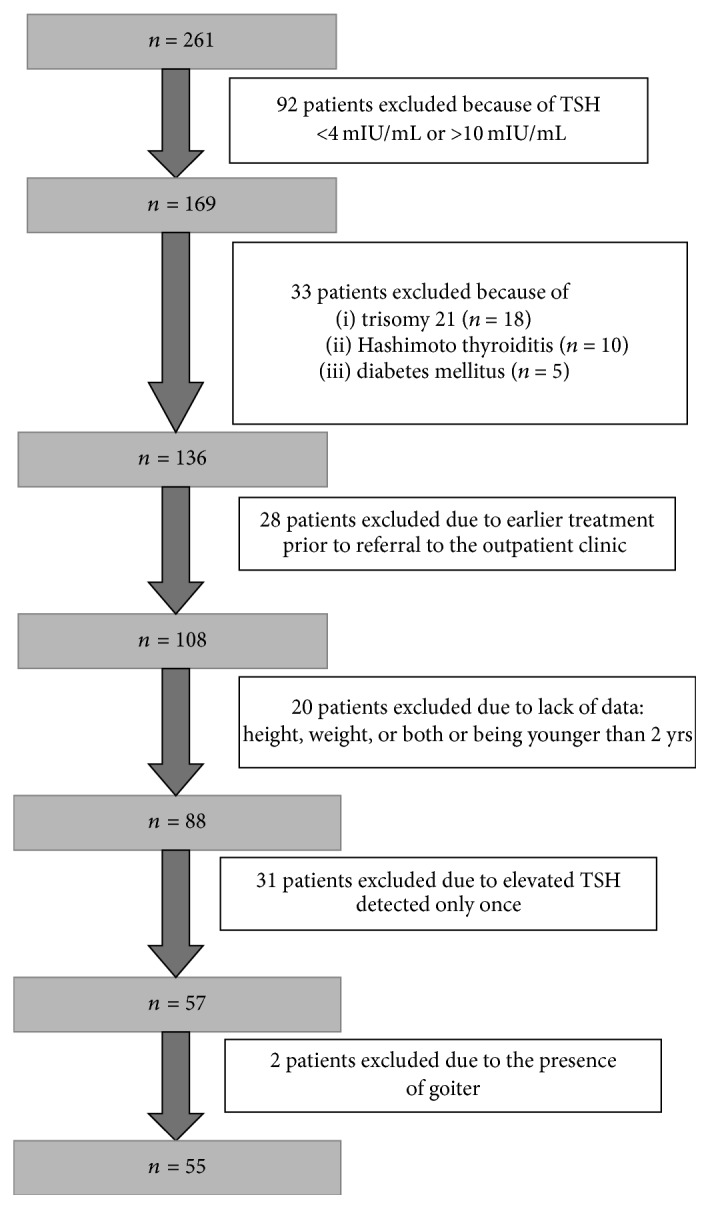
Exclusion criteria: patients referred to the Outpatient Clinic with diagnosis of sHT.

The process of patients' selection is presented in Figure 1. The final study group consisted of 55 children and adolescents who presented serum TSH concentrations between 4.0 and 10.0 mIU/mL, with fT4 and fT3 within their reference ranges (group-sHT). The median age in the sHT group was 9.92 years, mean 9.45 ± 4.25 years (range from 2.0 to 17.33 years). The median TSH concentration was 5.51 mIU/mL, mean 5.67 ± 1.20 mIU/mL (range from 4.00 to 9.40). Positive family history towards thyroid gland disease was reported in 4 (7.30%) patients. At least one symptom suggesting hypothyroidism was observed in 44 (80.0%) children. The BMI *Z*-score was 0.30 ± 1.64, and the hSDS was −0.35 ± 1.79. The baseline comparison between girls and boys showed no significant differences regarding age, mean TSH level concentration, positive family history, mean BMI *Z*-score, or hSDS. Detailed characteristics are presented in Table 1.

Out of 55 sHT patients, 33 (60.0%) started L-thyroxine therapy.  Table 2 shows the baseline comparison between patients who received treatment (sHT-T1) and those who did not (sHT-T0). There were no statistically significant differences between the groups regarding the mean age at the first visit (range from 2.87 to 15.6, median age 9.95 years versus range from 2.0 to 17.33, median age 9.62 years, T1 and T0, resp.), BMI *Z*-score, and hSDS values. T1 patients had higher serum TSH concentrations than T0 patients, but the difference was not statistically significant (*p* = 0.09). The two groups differed with regard to the length of follow-up (*p* < 0.01). Four (11.8%) children in group T1 and none in group T0 had a positive family history of thyroid disorders. The patients' clinical presentations detected at the first examination are presented in Table 3. Nine (27.3%) patients in group T1 and five (22.7%) in T0 showed more than one clinical symptom suggesting hypothyroidism.

The mean cumulative dose of L-thyroxine used in 33 patients was 0.82 ± 0.48 *μ*g/kg/day, median dose: 0.71 *μ*g/kg/day, range from 0.25 *μ*g/kg/day to 2.41 *μ*g/kg/day.

During the observation period 15 (68.2%) children and adolescents without treatment became euthyroid (TSH < 4.0 mIU/mL): 11 within three months from the last determination of TSH level, 1 at 3–6 months, and 3 at 6–12 months. In 7 (31.8%) patients, the TSH concentration remained above the reference range but under <10.0 mIU/mL at the end of follow-up.

In our study, 4 (7.3%) patients (3 girls and 1 boy) had baseline TSH levels over 7.5 mIU/mL, of whom only 1 showed progression to overt hypothyroidism. The patient, a 5.5-year-old girl, had baseline TSH of 8.14 mIU/mL, positive family history towards thyroid gland disease, and a BMI *Z*-score of 1.84. The length of follow-up was 1,453 days. The treatment involved a cumulative L-thyroxine dose of 1.06 *μ*g/kg/day.

We identified a group of 31 children and adolescents, with a mean age of 8.7 ± 4.24 years, in whom an elevated TSH serum concentration was detected only once (non-sHT group). During the second/control measurement in this group, the TSH concentration was within its reference range. Non-sHT patients were then compared with patients with confirmed subclinical hypothyroidism (sHT group) (Table 4). The non-sHT patients were referred to the outpatient clinic due to abnormal TSH results (range from 4.0 to 8.73 mIU/mL) with or without symptoms suggesting hypothyroidism and without history of an active or recent acute illness that could cause a transient drop in thyroid hormone production. At baseline, the sHT and non-sHT groups did not differ with regard to age, BMI *Z*-score, hSDS, or positive family history; however, serum TSH was significantly lower in non-sHT patients (Table 4).

## 4. Discussion

Our retrospective study aimed to evaluate the course of sHT in children and adolescents and to establish the rationale for substitutional therapy induction in this group of patients. The results of our analysis showed that there was no clear clinical or laboratory explanation as to why treatment was initiated in young patients with subclinical hypothyroidism. Positive family history seemed to be the only discriminant; sHT patients with L-thyroxine therapy more frequently had family members with diagnosed thyroid disorders. Despite the small size of the study group, we confirmed a low incidence of conversion of sHT to overt hypothyroidism.

### 4.1. Prevalence of sHT

According to the literature, the prevalence of subclinical hypothyroidism in the young population is estimated to be less than 2% [3]. In our study, out of 261 patients referred to the outpatient clinic between 2009 and 2014 with suspicion of subclinical hypothyroidism; only 55 (21.7%) children and adolescents met the criteria of sHT. The obtained results cannot be generalized to the entire population of Silesian children as the data came from a large but single clinical center.

### 4.2. The Upper Limit of Reference Range of TSH

The cut-off point of TSH concentration at which pediatricians should diagnose their patients with subclinical hypothyroidism has not as yet been determined. In our center, the diagnosis is made when the TSH level is at least 4.0 mIU/mL (reference limit), regardless of age. The cut-off point in the study by Lazar et al. [12] was 5.5 mIU/mL, whilst Wasniewska et al. [13] adopted a cut-off point of 5.0 mIU/mL. Hamilton et al. [14] found the upper reference limit of TSH level of 4.1 mIU/mL to be more compatible with clinical experience and a reasonable compromise.

### 4.3. sHT and Symptoms

The symptoms of hypothyroidism are neither sensitive nor specific. Indeed, patients in a euthyroid state are difficult to distinguish from patients with thyroid hormone deficiency because the clinical manifestation is affected by disease duration, its severity, and individual sensitivity to thyroid hormone deficiency. Patients who report many or newly developed symptoms are most likely to have subclinical or overt hypothyroidism. In their review, Gawlik et al. [15] presented several studies, in which sHT either was confirmed as a result of clinical presentation, or was diagnosed incidentally, and only then assessed clinically for typical manifestation.

The data available in the literature are still controversial regarding the indications and effects of substitution therapy on improving the growth status of children with short stature and subclinical hypothyroidism. In their prospective study of 39 patients with short stature and sHT, Cetinkaya et al. [16] showed that both prepubertal and pubertal patients experienced a significant improvement in growth after 6–12 months of treatment with L-thyroxine.

Cerbone et al. [17] conducted a cross-sectional controlled study involving 36 children with sHT who had never been treated with L-thyroxine and 36 age- and sex-matched healthy children. The patients were followed longitudinally for a mean of 3.3 years, revealing that untreated long-standing sHT was not associated with deterioration in growth and bone maturation. Moreover, all the recruited children with sHT and short stature were diagnosed with familial short stature and/or possible constitutional delay of growth and puberty. Therefore, when deciding on treatment modality in children with SH and short stature, the expected benefits as well as side effects of L-thyroxine and the possibility of iatrogenic hyperthyroidism should be considered.

The recommendations concerning overweight young patients with sHT are more precise: pharmacological treatment should be avoided, since moderately elevated levels of TSH are a consequence rather than a cause of overweight. In obese children with sHT, first, a dietary-behavioral management intervention should be recommended in order to reduce weight, irrespective of the use of levothyroxine [18–20]. Other nonpathognomonic symptoms were only reported by single patients, which raises the question whether subclinical hypothyroidism actually causes symptoms or whether their presence is a coincidence.

In our study, the vast majority of patients 44/55 (80.0%) showed at least one nonpathognomonic symptom, some of them suggesting hypothyroidism. Analyzing overweight, obesity, and short stature, we noticed a similar incidence between patients who were referred for LT-4 therapy and those who were not. The type of symptoms did not affect the decision whether to initiate treatment.

### 4.4. Treatment with L-Thyroxine

There are no clear guidelines on the management of subclinical hypothyroidism in young patients, the frequency of TSH measurements, and the length of follow-up in patients without therapy. According to the European Thyroid Association Guidelines for the Management of Subclinical Hypothyroidism in Pregnancy and in Children for 2014, the decision whether or not to treat should be made after discussing with the parents the side effects and risks as well as the potential benefits of therapy with L-thyroxine. The 2014 cross-sectional, controlled study by Cerbone et al. [5] showed that untreated mild long-lasting idiopathic sHT may be associated with subtle proatherogenic disturbances, although it is difficult to establish whether such mild abnormalities represent the early steps in the initiation of atherogenesis. Currently, there is little evidence to recommend treatment in most children with sHT in whom serum TSH is <10.0 mIU/mL, with fT4 within its normal range [21]. In his review, Kaplowitz [22] showed that a pediatric endocrinologist may decide to start thyroid hormone therapy immediately after confirmation of elevated TSH, or may recommend frequent monitoring of TSH for prolonged periods, or may suggest that, unless a follow-up test shows a further significant rise in TSH or a subnormal free T4 concentration, no action is required.

Bona et al. [23] concluded that sHT in children and adolescents is often a self-remitting process and its treatment should be considered only when TSH values are higher than 10 mIU/mL, when clinical signs or symptoms of impaired thyroid function or goiter are detected, or when sHT is associated with other chronic diseases.

Our study aimed to analyze the management of patients diagnosed with sHT and to establish the factors that encouraged physicians to prescribe thyroid hormones. There were no differences with regard to age at baseline, TSH level, BMI *Z*-score, hSDS, and presence of clinical symptoms suggesting hypothyroidism between patients who underwent therapy with LT-4 and those who did not. Our study shows that the pediatric endocrinologists in our clinical center made subjective decisions whether or not to treat, relying only on their own experience and, possibly, on positive family history towards thyroid disease.

The mean dosage of L-thyroxine applied in our patients, sufficient to maintain TSH within its normal ranges, was 0.82 ± 0.48 *μ*g/kg/day; the lowest dose was 0.25 *μ*g/kg/day and the highest was 2.41 *μ*g/kg/day. In the first prospective study conducted by Wasniewska et al. [13] in 92 children with LT-4, the initial dose was 2.0 *μ*g/kg/day and was further adjusted on the basis of fT4 and TSH serum concentrations. In her review, Bona et al. [23] mentioned five articles [16, 24–27] regarding replacement therapy with LT-4, in which the mean dosage of LT-4 varied from 2 to 4 *μ*g/kg/day [24–26]. In two studies [16, 27], information on the dose of levothyroxine was not provided. These data confirm the lack of clearly established guidelines as to what dosage of LT-4 may protect children from proceeding to overt hypothyroidism. The dosage of LT-4 lower than 2 *μ*g/kg/day administered to our patients was sufficient to maintain TSH in its reference range.

### 4.5. Normalization of TSH Level

In our study, a vast majority (68.2%) of patients without therapy with L-thyroxine normalized their TSH concentration and became euthyroid during their follow-up period. At the end of follow-up, none of the patients showed a highly elevated TSH serum level above 10 mIU/mL, though 31.8% still had TSH above the upper limit of the reference range. Similar results were presented by Wasniewska et al. [28]: 41.3% of patients normalized their TSH at the end of a 2-year follow-up period. Out of the remaining patients 11.9% had a further increase in TSH over 10 mIU/mL. Based on the obtained results, the authors concluded that there was a progressive decrease of the natural course of SH.

In another recently published longitudinal study, Wasniewska et al. [29] assessed the natural course of sHT in 127 girls according to the etiology of subclinical thyroid disease. At the end of the 5-year follow-up, girls diagnosed with idiopathic sHT had a higher rate of normalization of TSH levels than girls with sHT related to Hashimoto's thyroiditis (61.9% versus 10.6%).

Our study raises the following questions: how often should TSH concentrations be determined in order to accurately and reliably evaluate the course of sHT? How long should the follow-up of patients after normalization of TSH last? Does it depend on family history? The child's wellbeing must always be considered. Multiple TSH determinations are associated with stress and discomfort connected with blood collection and medical visits. Thus, establishing the exact frequency of determining thyroid gland parameters seems paramount. The economic aspect should not be disregarded either: too frequent and unnecessary visits generate costs, which is a serious limiting factor in health care. Moreover, TSH assessment must be performed in appropriate measurement conditions (without infection symptoms, etc.) and cannot be used as a routine laboratory test.

### 4.6. Progression to Overt Hypothyroidism

In our study, out of the 55 patients who met the criteria for sHT, only 1 (1.8%) girl proceeded to overt hypothyroidism. According to the longitudinal study conducted by Lazar et al. [12], out of 121,052 children and adolescents, less than 1% proceed to overt thyroid dysfunction, either hypothyroidism or hyperthyroidism. Baseline TSH level above 7.5 mIU/mL and female gender are risk factors for developing overt hypothyroidism, which is consistent with our findings. According to Wasniewska et al. [29], girls with Turner syndrome or trisomy 21 with sHT related to Hashimoto's thyroiditis are at a higher risk of proceeding to overt hypothyroidism than girls with isolated or idiopathic sHT. Due to the small size of the group, no definitive conclusions can be drawn.

### 4.7. Limitations of Our Study

The main limitation of our study was its retrospective character and the small number of patients; however our center is the largest one in the southern Poland. Only one patient during the follow-up developed an overt thyroid dysfunction; therefore the survival analysis was not performed in this study. The study with its limitations was aimed at being another contribution to the debate on the desirability of creation the algorithms for appropriate care of patients with SH.

## 5. Conclusions

Further prospective, longitudinal, and case-controlled studies are warranted in order to establish final treatment indications, if any, and the recommended mean dosage of LT-4. The optimal dose should on the one hand prevent children and adolescents with sHT from proceeding to overt thyroid disease and on the other hand not cause an excess of thyroid hormones, which may affect the body's metabolic processes. To this end, our center has just begun a 2-year prospective controlled study approved by the Ethics Committee.

## Figures and Tables

**Table 1:** Characteristics of the study group by gender (baseline data).

	Girls (*n* = 31)	Boys (*n* = 24)	*p* value
Age, mean	10.02 ± 4.32	8.72 ± 4.15	**0.33**
BMI *Z*-score, mean	0.36 ± 1.76	0.23 ± 1.51	**0.78**
hSDS, mean	−0.29 ± 1.84	−0.43 ± 1.75	**0.78**
TSH, mean	5.79 ± 1.34	5.51 ± 1.0	**0.39**
FU, mean (months)	15.78 ± 14.58	13.38 ± 12.30	**0.58**
Positive family history (%)	2 (6.25)	2 (8.0)	**0.79**
Presence of symptoms (%)	27 (84.4)	17 (68.0)	**0.16**

BMI *Z*-score: body mass index standard deviation score; hSDS: height standard deviation score; FU: follow-up.

**Table 2:** Baseline comparison of patients treated with LT-4 (group T1) and patients without treatment (group T0).

	Group T1 (*n* = 33)	Group T0 (*n* = 22)	*p* value
Age, mean	9.75 ± 4.0	9.0 ± 4.67	**0.52**
BMI *Z*-score, mean	0.12 ± 1.57	0.58 ± 1.75	**0.16**
hSDS, mean	−0.32 ± 1.89	−0.38 ± 1.66	**0.80**
TSH, mean	5.9 ± 1.36	5.33 ± 0.85	**0.09**
FU, mean (months)	20.66 ± 13.08	5.85 ± 8.63	**<0.01**
Positive family history (%)	4 (11.8)	0 (0)	**<0.01**
Presence of symptoms (%)	25 (73.5)	19 (86.4)	**0.12**

BMI *Z*-score: body mass index standard deviation score; hSDS: height standard deviation score; FU: follow-up.

**Table 3:** Patients' complaints/features at first examination.

	Group T0 (*n* = 22)	Group T1 (*n* = 33)	*p* value
Obesity (%)	6 (27.3)	2 (6.1)	**0.07**
Overweight (%)	6 (27.3)	10 (30.3)	**0.92**
Weight deficiency (%)	3 (13.6)	4 (12.1)	**0.81**
Significant weight deficiency (%)	3 (13.6)	3 (9.1)	**0.93**
Short stature (%)	4 (18.2)	6 (18.2)	**0.72**
Hair loss (%)	1 (4.5)	0 (0.0)	**0.84**
Constipation (%)	1 (4.5)	0 (0.0)	**0.84**
Impaired concentration (%)	0 (0.0)	2 (6.1)	**0.66**
Cold intolerance (%)	0 (0.0)	3 (9.1)	**0.39**
Menstrual disorder (%)	1 (4.5)	1 (3.0)	**0.66**
Chronic fatigue (%)	0 (0.0)	1 (3.0)	**0.84**
Dry skin (%)	1 (4.5)	5 (15.2)	**0.43**

**Table 4:** Comparison of patients with elevated TSH detected only once (group non-sHT) and patients followed up for a long time (group-sHT)—baseline data.

	Group-sHT (*n* = 55)	Group non-sHT (*n* = 31)	*p* value
Age, mean	9.45 ± 4.25	8.7 ± 4.24	**0.43**
BMI *Z*-score, mean	0.30 ± 1.64	0.84 ± 1.48	**0.09**
hSDS, mean	−0.35 ± 1.79	0.15 ± 1.37	**0.19**
TSH, mean	5.67 ± 1.2	5.1 ± 1.16	**0.02**
Positive family history (%)	4 (7.3)	5 (16.1)	**0.32**
Presence of symptoms (%)	44 (80.0)	12 (38.7)	**0.01**

BMI *Z*-score: body mass index standard deviation score; hSDS: height standard deviation score.
